# Potential Use of Elderberry (*Sambucus nigra* L.) as Natural Colorant and Antioxidant in the Food Industry. A Review

**DOI:** 10.3390/foods10112713

**Published:** 2021-11-05

**Authors:** Rubén Domínguez, Mirian Pateiro, Paulo E. S. Munekata, Eva María Santos López, José Antonio Rodríguez, Lillian Barros, José M. Lorenzo

**Affiliations:** 1 Centro Tecnológico de la Carne de Galicia, 32900 San Cibrao das Viñas, Spain; mirianpateiro@ceteca.net (M.P.); paulosichetti@ceteca.net (P.E.S.M.); jmlorenzo@ceteca.net (J.M.L.); 2 Área Académica de Química, Universidad Autónoma del Estado de Hidalgo, Ctra. Pachuca-Tulancingo Km 4.5 s/n, Col. Carboneras, Mineral de la Reforma 42183, Hidalgo, Mexico; emsantos@uaeh.edu.mx (E.M.S.L.); josear@uaeh.edu.mx (J.A.R.); 3 Centro de Investigação de Montanha (CIMO), Instituto Politécnico de Bragança, 5300-253 Bragança, Portugal; lillian@ipb.pt; 4 Área de Tecnología de los Alimentos, Facultad de Ciencias de Ourense, Universidad de Vigo, 32004 Ourense, Spain

**Keywords:** food natural additives, natural dies, synthetic additives replacer, anthocyanins, antioxidants, black elder, European elder, berry

## Abstract

The food industry, in response to current consumer demand for natural and functional foods, is constantly evolving and reformulating traditional products formulations. Thus, during the last decades, multiple natural sources have been investigated to replace the need to add synthetic additives. In addition, the use of natural sources can also increase the nutritional quality of the food. With this in mind, elderberry is used in the food industry for certain purposes. However, its potential is much higher than the number of applications it currently has. Its high content of anthocyanins, as well as other polyphenols and vitamins, means that it can be used by the food industry both as a colorant and as an antioxidant. In addition, the incorporation of these bioactive compounds results in functional foods, with a high antioxidant capacity. Moreover, the inclusion of elderberry products in foods formulation increases their shelf-life, but the correct amount and strategy for adding elderberry to food should be studied to ensure a positive effect on nutritional and technological properties without affecting (or improving) the sensory quality of foods. Therefore, this manuscript aims to review the main bioactive compounds present in elderberries, as well as their potential uses in the food industry.

## 1. Introduction

The food industry is of vital importance both from a social and economic point of view [1]. The intake of nutritious food ensures both health and correct human nutrition. In this sense, it is important to highlight that foods are susceptible to degradation processes. Among these processes, oxidative reactions are one of the most important since they generate several toxic and harmful compounds and also decrease the nutritional properties (losses of important nutrients) and sensory quality (appearance of off-flavors, discoloration, etc.) of foods during storage [2]. Furthermore, it is well known that the visual aspect, and more specifically the color, is one of the attributes that largely determines the purchase intention of the consumers [3], but color suffers modifications during food storage. These facts decrease the shelf-life of foods and cause consumer rejection and high economic losses in the food industry. Consequently, for several decades, synthetic additives such as antioxidants have been used to delay the degradative reactions [4]. In order to maintain a food’s characteristic color, the use of colorants is also common in the food industry [1]. However, several studies have indicated a relationship between the intake synthetic additives and health issues [5] due to their potential toxicity [6] and carcinogenic implications [7]. As a result, current consumers are aware of the relationship between diet and health, and they demand more natural and healthy products. Therefore, in recent decades, the food industry has tried to find new and sustainable natural sources of additives, such as plant extracts, as alternatives to synthetic additives [1,8,9,10,11]. Among all natural water-soluble pigments, the anthocyanins that are present in several berries are promising additives that possess both important antioxidant activity and intense color [12,13,14]. These compounds (anthocyanins) are a subgroup of flavonoids that contribute to the blue, purple, and red colors in many fruits [15,16].

**Figure 1:**
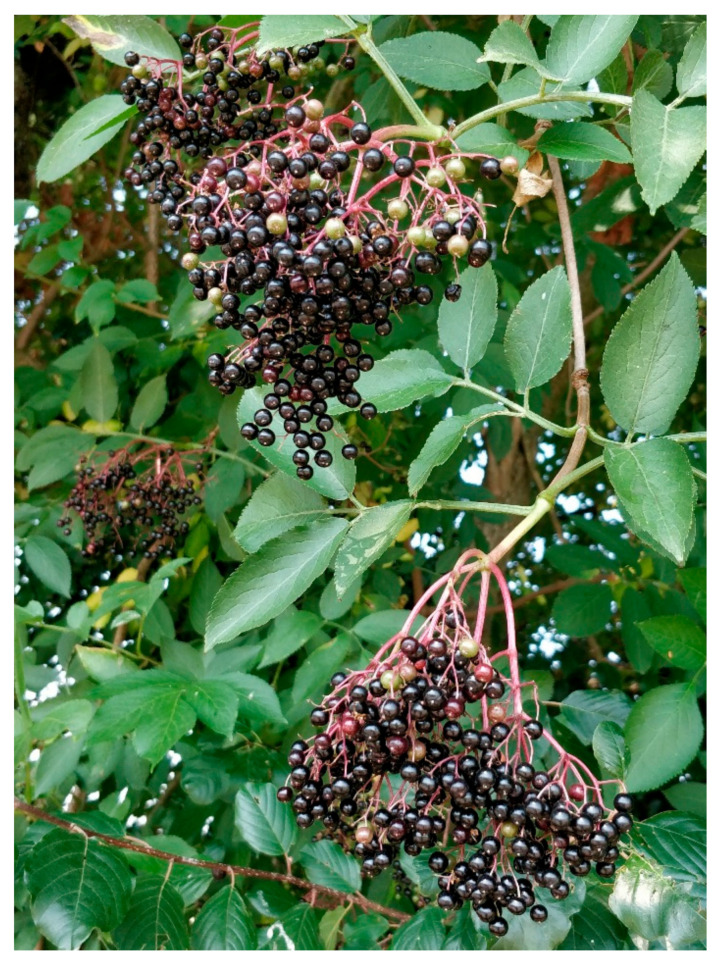
Example of wild elderberries on the tree.

In recent years, elderberry (*Sambucus nigra L.*) has been found to be an important anthocyanin-rich berry (Figure 1). The elderberry and its preparations (extracts, powders, etc.), which have high anthocyanin content, mainly cyanidin glycosides [17], can be used in many foods as coloring agents [18,19,20]. Thus, there is a growing interest in the use of elderberries as a functional ingredient and natural additive in the manufacture of food products [17]. It has been used as a medicinal plant in folk medicine for several centuries since anthocyanins are known phytochemicals with human health benefits [16]. Elderberries have been used to treat flu and to stimulate the immune system [15,21,22]. In addition, these berries have significant antioxidant, anticarcinogenic, anti-inflammatory, antimicrobial, anticonvulsant, antidiabetic, hypocholesterolemic, and antiviral effects and have shown immunomodulatory and antidepressant activity [14,15,23,24,25]. Consequently, several beverages and also food supplements have been developed [15,21]. However, it should be noted that the elderberries also contain cyanogenic glycosides, which are mildly poisonous, causing vomiting, although this toxicity could be overcome by cooking [15,26]. The most abundant cyanogenic glucosides are sambunigrin, prunasin, m-hydroxysubstituted glycosides, zierin, and holocalin [26,27,28]. Even so, the berries have the lowest values of these toxic compounds in comparison with the cyanogenic glucosides content in leaves or flowers [26].

Additionally, the content in bioactive compounds also depends on different parameters and differs between wild edible plants and cultivated elderberries or between varieties [29,30,31,32], location, growing season, ripening stage, and climatic conditions [26,33]. While these factors could affect their composition, elderberries possess a high potential since they have higher total phenolic and anthocyanins content and antioxidant activity than other important berries, such as blackcurrant and raspberry [34]. Several manuscripts were published about the use of elderberries in the medical field, and this aspect suggests that this topic is widely studied and that the properties of elderberries have been demonstrated. In fact, as previously mentioned, important antiviral and immunomodulatory effects have been attributed to elderberries in recent research [35,36,37,38]. Therefore, although elderberries are normally used in the pharmaceutical industry due to their positive health benefits [15,21], their characteristics also make them suitable for food purposes, and their anthocyanins could be used as natural coloring agents while providing positive effects to consumers’ wellbeing [6]. However, elderberries are underutilized and are not commonly used as food ingredients [23], and they are still rarely applied in food formulations [19]. One of the possible reasons behind this fact could be related to the challenge of introducing anthocyanins into foods. This is because of their low stability under some conditions, such as light exposure, oxygen exposure, thermal treatments, pH changes, etc. [39]. However, these are common situations during food processing, forcing researchers to find new techniques to stabilize anthocyanins. In this regard, various recent studies investigated the optimal conditions for obtaining high anthocyanins extracts from elderberries [14,34] and also explored their stabilization using encapsulation processes [18,34,40]. In another study, the authors also proposed the production of pomace powder from elderberry and its application as a coloring foodstuff [39].

Therefore, and taking into account the previously mentioned enormous benefits that elderberries possess, this manuscript reviews the possible uses of these berries to obtain natural antioxidants and coloring compounds or extracts and their potential use in the food industry. This review also conducted an exhaustive search on the main bioactive compounds (both antioxidants and colorants) present in elderberries.

## 2. Bioactive Compounds in Elderberries

Elderberry (*Sambucus nigra* L.) is a good source of several important and valuable compounds. It is rich in basic nutrients, such as carbohydrates (~18%) (mainly simple sugars [15,41]), fiber (~7%), proteins (~3%), and lipids (0.35%) (mainly in seeds; ~22%) [14,26,42,43]. In lipids, they present an exceptional composition, with very high polyunsaturated fatty acids amounts (~80%), low amounts of saturated fatty acids (<10%), and with an n-6/n-3 ratio of about 1 [14], which agrees with all health institutions’ recommendations for healthy fat/oil ratio. Additionally, the content of essential fatty acids is very high [14,41]. Linoleic acid (C18:2n-6; an n-6 fatty acid) is the major fatty acid in elderberries, representing 39% of total fatty acids, followed by α-linolenic fatty acid (C18:3n-3; an n-3 fatty acid) with similar values (38%) [14]. Both fatty acids are essential; thus, the sum of essential fatty acids in elderberries represents more than 77% of total fat [14].

Regarding micronutrients, elderberries also contain important amounts of vitamins, antioxidants, and minerals (mainly magnesium and calcium) [26,42,44]. Vitamin C represents ~1700 µg/g of dry elderberries (DW), β-carotene (pro-vitamin A) ~18 µg/g DW, and total tocopherols (vitamin E) 324 µg/g DW [41], mainly α-tocopherol (~300 µg/g DW). Similar results were reported by other researchers, who concluded that elderberry seed flour is a source of α-tocopherol, which has the highest vitamin E bioactivity, as well as γ-tocopherol, which shows better antioxidant potential [26]. Additionally, B complex vitamins (B2, B3, B5, B6, B9) were found in elderberries [15,43].

It is well known that vitamins E and C are important natural antioxidants; thus, the content of these compounds partially contributes to the antioxidant activity of elderberries. In fact, α-tocopherol (vitamin E) is considered one of the most effective antioxidants [2] because it transfers hydrogen atoms to radicals, delays hydroperoxides decomposition, scavenges singlet oxygen and metals complexes in the presence of ascorbate. In addition, vitamin C (ascorbic acid) also has a positive effect on the antioxidant activity of α-tocopherol since it neutralizes tocopheroxy radicals and regenerates the α-tocopherol molecule [2]. Thus, the presence of both vitamin E and C produce a synergic antioxidant effect. Furthermore, vitamin C can scavenge oxygen and various free radicals [2]. Finally, pro-vitamin A (β-carotene) has both antioxidant and colorant properties. Regarding its antioxidant capacity, β-carotene scavenges radicals, resulting in a carbon-centered radical that is stabilized by resonance [2]. However, due to its low content, the importance of β-carotene in the total antioxidant and colorant properties of elderberries is insignificant in comparison with other compounds.

**Figure 2:**
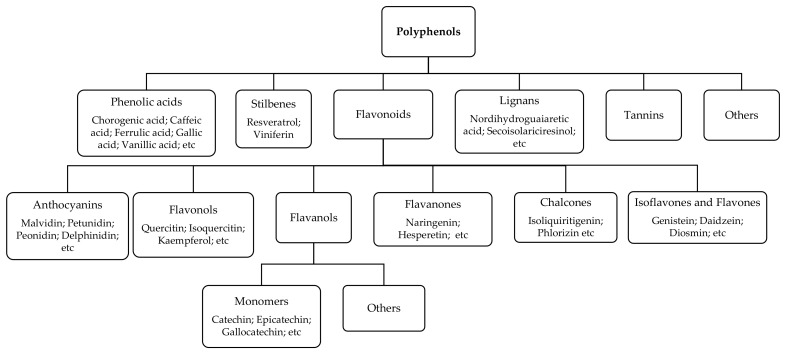
Polyphenols classification based on the number of phenol rings and their structural elements (adapted from Lopez-Fernandez et al. [45]).

However, the most important bioactive compounds that contribute to the antioxidant properties of elderberries are polyphenols. Polyphenols are secondary plant metabolites constituted by several molecules, which can be classified according to their chemical structure based on the number of phenol rings and their structural elements [45] (Figure 2).

Among the main polyphenols in elderberries, the antioxidant properties of this fruit are mostly determined by anthocyanins, as well as by flavonols and phenolic acids [33] (Table 1). Flavonoids, which include both anthocyanins and flavonols, are the most abundant polyphenol group in elderberries [17]. Regarding phenolic acids, chlorogenic acid occurs in the highest concentrations [17,44]. However, other important acids were found in this fruit, such as sinapic acid, t-cinnamic acid, neochlorogenic acid, and crypto-chlorogenic acid [26,30,33,42]. In addition to the aforementioned phenolic acids, gallic, gentisic, vanillic, 4-hydroxybenzoic, coumaric, and ferulic acids [14] and syringic, benzoic, and rosmarinic acids [33] were also found in elderberries.

Flavonols are other important polyphenols in elderberries. As commented below, several flavonol molecules were observed in elderberries, but rutin (quercetin 3-rutinoside) and quercetin were the dominant flavonols [14,17,33]. Among them, the rutin content was higher than that of quercetin [14,33]. Thus, quercetin and its derivatives occur in the highest concentrations [44]. In addition to these compounds, other flavonols were also found, such as quercetin-3-glucoside, kaempferol-3-rutinoside, kaempferol-3-glucoside, isorhamnetin-3-rutinoside, and isorhamnetin-3-glucoside [42]. However, it is important to highlight that these other flavonols occur in elderberries in smaller amounts [26].

Finally, the major phenolic compounds in elderberries are anthocyanins, present mostly as cyanidin derivatives [28,29,33]. Among all of the anthocyanins, there is substantial cyanidin 3-sambubioside and cyanidin 3-glucoside content [33,46]. Nevertheless, there is also an important amount of cyanidin 3-sambubioside-5-glucoside and cyanidin 3,5-diglucoside content [15,43]. Some authors reported that the most abundant anthocyanin in elderberries was cyanidin 3-sambubioside (>50% of total anthocyanins) [25], also found in the Samocco cultivar [31], while in other studies, cyanidin 3-glucoside was reported as the main anthocyanin in the Korsør and Haschberg cultivars (50–65%) [30,31,33]. In any case, both anthocyanins (cyanidin 3-sambubioside and cyanidin 3-glucoside) are the two major anthocyanins in elderberry, contributing together around 85–90% of the total anthocyanins present in the fruit [18,30,47,48].

**Table 1:** Main polyphenols in elderberries (phenolic acids, flavonols, and anthocyanins).

Polyphenols		
Phenolic Acids	Flavonols	Anthocyanins
Chlorogenic acid (5-caffeoylquinic acid)	Rutin (quercetin 3-rutinoside)	Cyanidin 3-sambubioside
Neochlorogenic acid (3-caffeoylquinic acid)	Quercetin	Cyanidin 3-glucoside
Cryptochlorogenic acid (4-caffeoylquinic acid)	Isoquercitrin (quercetin 3-glucoside)	Cyanidin 3-sambubioside-5-glucoside
Ferulic acid	Quercetin 3-galactoside	Cyanidin 3,5-diglucoside
Coumaric acid	Quercetin 3-hexoside	Cyanidin 3-rutinoside
Vanillic acid	Quercetin 3-vicianoside	Cyanidin 3-coumaroyl-sambubioside
4-Hydroxibenzoic acid	Quercetin 3-(6″-acetyl) galactoside	Cyanidin 3-coumaroyl-glucoside
Gallic acid	Kaempferol 3-rutinoside	Cyanidin 3-coumaroyl-sambubioside-5-glucoside
Gentisic acid	Kaempferol 3-glucoside	Cyanidin
Sinapic acid	Kaempferol	Malvidin
Cafeic acid	Catechin	Pelargonidin 3-glucoside
Quinic acid	Epicatechin	Pelargonidin 3-sambubioside
Benzoic acid	Myricetin 3-rutinoside	Delphinidin 3-rutinoside
Syringic acid	Isorhamnetin 3-rutinoside	Peonidin 3-arabinoside
t-Cinnamic acid	Isorhamnetin 3-glucoside	Cyanidin-xylosyl-rutinoside

Data obtained from published studies [6,14,17,22,26,28,29,30,32,33,47,48].

In contrast with these findings, some authors reported that cyanidin 3-sambubioside-5-glucoside had higher values (36.4%) than cyanidin 3-glucoside (<20%) [3], while in American elderberry (*Sambucus canadensis*), cyanidin 3-(trans)-coumaroyl-sambubioside-5-glucoside (65–70%) [49] and cyanidin 3-sambubioside-5-glucoside (~50%) [32] were the most abundant anthocyanins. Nevertheless, it is clear that cyanidin 3-sambubioside, cyanidin 3-glucoside, and cyanidin 3-sambubioside-5-O-glucoside are the most important anthocyanins in elderberries (*Sambucus nigra* L.). In addition, in elderberries were also found minor quantities of cyanidin-3-rutinoside, pelargonidin-3-glucoside, delphinidin-3-rutinoside, cyanidin 3-coumaroyl-sambubioside, or cyanidin 3-coumaroyl-glucoside [26].

**Figure 3:**
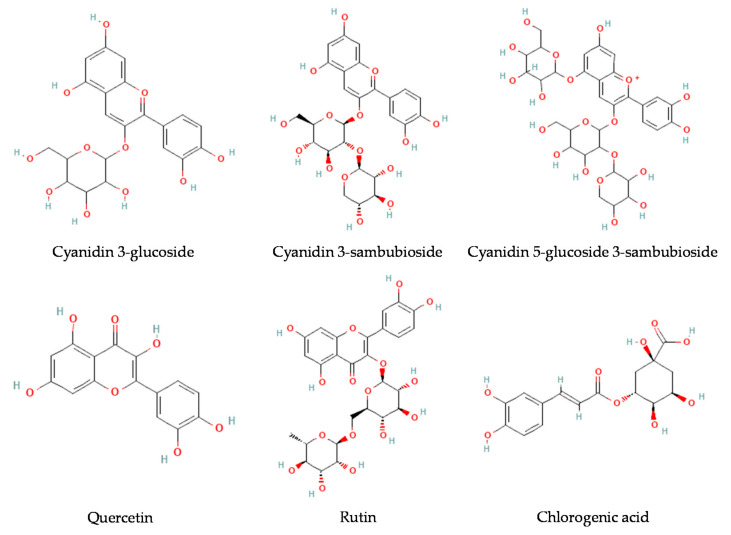
Main bioactive molecules of elderberries.

As a general conclusion, there are several compounds that contribute to the antioxidant and colorant properties of elderberries and their products (e.g., extracts, powders, juice), including multiple polyphenols and vitamins. However, taking into account their high content, it can be stated that among all these compounds, the antioxidant and coloring properties are directly related to the content of the five most abundant compounds, which include three anthocyanins (cyanidin 3-sambubioside, cyanidin 3-glucoside, and cyanidin 3-sambubioside-5-glucoside), two flavonols (rutin and quercetin), and one phenolic acid (chlorogenic acid) (Figure 3).

In addition to the antioxidant properties of anthocyanins, they are also natural pigments. Consequently, elderberries are used as natural colorants in different industries. However, the typical color of these compounds varies with the pH. The elderberries’ anthocyanins exhibit red hues at acidic and blue hues at alkaline pH [49]. Particularly, elderberry is used as a food colorant and in pharmaceuticals [26]. The application of elderberries in the food industry (as colorant and antioxidant) is discussed in the next section.

## 3. Application of Elderberries in the Food Industry

**Figure 4:**
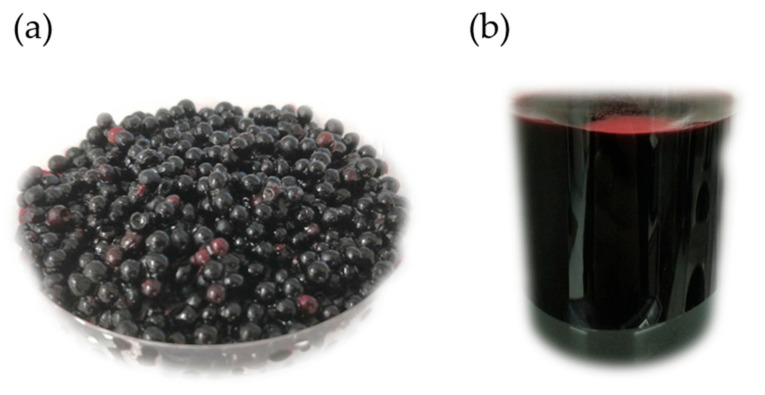
Ripe fresh elderberries for the extraction of bioactive compounds (**a**) and typical dark red color of the extract (**b**) obtained with a hydroalcoholic solution (acidic pH) using the Domínguez et al. procedure [14].

Berries are offered to the markets in processed form [15]. In fact, elderberries have been used in the food industry to produce pies, jellies, jams, ice creams, yoghurts, and alcoholic beverages [14,25,27]. Despite the fact that some studies presented very promising results and the excellent properties of the elderberries as a colorant (Figure 4) and antioxidant [14], the number of researchers who have investigated the use of this berry in foods has been very limited.

It is also important to highlight the restrictions or the cautions about the use of elderberries and their derived products (extracts, powders, juices, etc.) in foods. As previously mentioned, the applicability of anthocyanins as natural colorants is often hampered by the low stability towards heat, oxygen, light, and increased pH values [3,16]. Additionally, the storage conditions of elderberry could also exert an important effect on its antioxidant and colorant properties. In fact, in a recent study, the authors concluded that the content of total monomeric anthocyanins of elderberry juice concentrate stored at refrigerated temperatures (5 °C) was reduced by 14–22%, while in the same concentrates stored at room temperature the losses reached 67–71% [46]. Therefore, this section will introduce and discuss the main effects of the use of elderberry in the formulation of different foods and also as a new natural additive for use in the food industry.

According to published manuscripts, both the baking [6,50,51] and dairy [19,23,39,43] food industries use elderberries as natural additives to improve the color and shelf-life of their products. Likewise, although the application of elderberry products in the meat industry is scarce, the characteristic intense-red color of meats and meat products and their high perishability mean that elderberries can potentially be used in these products [14] due to the colorant and antioxidant properties that are discussed throughout this manuscript. Therefore, the meat industry would also be an industry in which the use of elderberry products would have great potential [14].

As can be seen in Table 2, there are several studies on the effect of elderberries on yoghurt. The first work investigated both the effect of the amount of elderberry juice added (10% or 25%), as well as the way to add it, either directly added juice or previously restructured juice, using alginate and sugar [43]. The first change was observed in color parameters. As expected, the characteristic red-purple of elderberry juice dramatically influenced the redness parameter (a*) of reformulated yoghurt. In this case, the addition of both—juice or restructured juice—produced significant changes, but these color modifications were more progressive in the yoghurts with restructured juice [43]. These authors attributed this behavior to the gradual release of juice from the alginate coating, and thus, the progressive release of anthocyanins into the yoghurt. The same trend was observed during storage (3 weeks). The storage did not produce modifications in a* values of yoghurts with restructured juice, while a significant increase was observed in yoghurts with the direct addition of juice. The presence of lactic acid bacteria, as well as the low pH of the yoghurts, may be the reason for the low dye stability of elderberry juice, but the restructured juice was more stable, which mitigated this degradation [43].

Changes were not only observed in color parameters since texture parameters also presented significant differences. The direct addition of juice at 10% did not produce any change in the yoghurt consistency, while when juice was added at 20%, the yoghurt fluidity increased [43]. In contrast, the restructured juice caused a 2-fold increase in yoghurt consistency. During storage, control yoghurt and yoghurt with restructured juice increased their viscosity, but this parameter decreased in the yoghurt reformulated with the direct addition of 20% elderberry juice. The progressive release of juice from restructured juice produced texture changes in comparison with the direct addition. With this in mind, it is clear that the presence of alginate in the restructured juice dominated the effect of the juice release, and therefore, the changes in both color and texture parameters [43].

Similarly, the high content of antioxidant compounds in elderberry juice produces a significant increase in antioxidant activity with both direct juice addition and restructured juice addition [43]. In this case, the juice content was an important parameter that influenced the antioxidant capacity. Additionally, the in vitro digestion of reformulated yoghurts also showed the high bioaccessibility of these antioxidants and an increase in antidiabetic activity (α-amylase and α-glucosidase), which are important aspects from a nutritional point of view [43].

Finally, according to a sensory evaluation, the yoghurts with restructured elderberry juice presented very high consumer acceptance (the most accepted), both at 0 days and after storage [43]. In contrast, the direct addition of juice resulted in yoghurts with low consumer acceptability, and more than 57% and 79% of consumers gave a dislike response for 10% and 20% reformulated yoghurts at day 0, respectively, while this rejection increased after storage (72–90% dislike) [43]. Therefore, and taking into account all changes, the best option in this study was the reformulation of yoghurts with 10% of restructured elderberry juice.

**Table 2:** Application of elderberries in the food industry.

Food Product	Elderberry Material	Amount	Main Effects	Ref.
Yoghurt	Elderberry juice and restructured elderberry juice ^1^	10% and 25%	Affected yoghurt color. The addition of restructured juice produced a more gradual change in the color of yoghurt than the direct addition of juice.	[43]
			Juice did not affect yoghurt consistency, but increased its fluidity. In contrast, restructured juice caused an increase in its consistency. The progressive release of juice from restructured juice produced texture changes.	
			The addition of elderberry produced an increase in the antioxidant capacity and the antidiabetic activity of yoghurt.	
			Restructured juice increased the sensory quality of yoghurt, while direct juice addition was less accepted by consumers.	
	Elderberry puree	10%	The addition of elderberry puree increased total solids, fiber, and carbohydrates, while it did not influence the rest of the chemical composition parameters of yoghurt.	[19]
			The elderberry yoghurt presented higher amounts of total phenolic, total anthocyanins, and antioxidant activity than natural yoghurt.	
			No differences were observed in the number of starter bacteria.	
			Texture parameters showed no significant differences.	
			The addition of elderberry puree increased redness (a*), while it decreased luminosity (L*) and yellowness (b*).	
			No differences were observed in sensory quality between control and elderberry yoghurt.	
	Elderberry pomace powder	2%	Total anthocyanin was higher than in those made with “reference substances” (black carrot and purple sweet potato), while their content remained stable during yoghurt storage (14 days).	[39]
			The total phenolic content increased slightly in elderberry yoghurts during storage.	
			Color saturation in elderberry pomace powder yoghurt did not change considerably in comparison with those formulated with commercial dye (black carrot).	
Kefir	Elderberry juice	10%	Commercial elderberry juice produced a lower increase in anthocyanins and phenolic compounds content than fresh elderberry juice. In fact, the fresh juice increased the content of both dramatically and also increased the antioxidant capacity of kefir.	[23]
			Sensory analysis showed that elderberry kefir sweetened with sucrose presented the highest consumer acceptability.	
Meat (charcoal-grilled pork loin)	Elderberry vinegar	3% (~30 mg/g meat)	Elderberry vinegar exhibited the highest polycyclic aromatic hydrocarbons (PAH) inhibition (82%). PAH formation involves free radicals generation; thus, the high polyphenols content of elderberry vinegar can act as an inhibitor of PAH formation through the elimination of free radicals precursors.	[47]
			Spraying meat with elderberry vinegar prior to grilling is an easy-to-apply strategy to limit the exposure to PAH.	
Meat (pork patties)	Elderberry powder	1%	Elderberry effectively decreased the formation of secondary oxidation products (thiobarbituric acid reactive substances; TBARs), and elderberry patties presented less than half TBARs values than control samples.	[52]
Gluten-free wafer sheets	Elderberry powder	1, 2, 3, 4 and 5%	The use of elderberry powder increased the bioactive compound (flavonoids) and mineral (iron, potassium, calcium, magnesium, and sodium) content in wafer sheets.	[50]
			The use of elderberry powder produced lower batter delamination than control wafer sheets, while it resulted in an increase in wafer breaking properties.	
			Elderberry reformulated wafers presented lower a*, b*, and L* values with the increasing amount of powder used.	
			The wafers elaborated with more elderberry powder (3–5%) had higher acceptable appearance scores, and the most acceptable gluten-free wafers could be obtained by adding 5% of elderberry powder.	
Croissants	Elderberry juice	2, 4 and 8%	The use of elderberry juice in a croissants formulation produced minimal changes in their nutritional quality.	[6]
			Reduction in L* and b* parameters, while an increase in a* was observed.	
			Elderberry croissants presented high antioxidant capacity and bioactive compounds content.	
Fiber-enriched pastas	Elderberry juice concentrate	-	The use of elderberry juice did not influence the processability of pasta.	[51]
			The elderberry addition produced a dark purple color pasta. After cooking, the pasta retained its purple color.	
			Elderberry pasta presented lower firmness values and increased the cooking loss.	
			The use of elderberries enhanced the pasta total polyphenol content. Additionally, the antioxidant activity of the reformulated pasta was also higher than the control.	
			The use of elderberries improved the nutritional value of pasta.	

^1^ Restructured elderberry juice produced by the mixture of 85% elderberry juice, 14% sugar and 1% of sodium alginate; a* the redness parameter; L* luminosity; b* the yellowness parameter.

In another study, the authors used 10% elderberry pure to reformulate probiotic yoghurts [19]. In this case, the use of elderberry puree had a very low influence on the yoghurt composition because the protein and fat content remained constant. However, the addition of puree induced positive nutritional changes because it increased the total solids, carbohydrates, and dietary fiber. The reformulation of probiotic yoghurt did not cause any differences in the starter bacteria viability, since both natural and elderberry pure yoghurts presented the same values. The authors attributed this fact to the prebiotic effect of elderberry fibers and the elimination of oxygen from the environment due to the actions of the antioxidant components, which enhanced probiotic survivability [19].

The content of total phenolic compounds and total anthocyanins content of reformulated yoghurts were extremely higher than natural yoghurt. This was expected since the high content of flavonoids (mainly anthocyanins) and other polyphenols in elderberry puree determined this increase. This fact resulted in a high antioxidant activity in the elderberry yoghurt (initially and after 29 storage days) since all these polyphenolic compounds are known as potent antioxidant substances [19].

As occurred in the previous study, the use of elderberry puree in probiotic yoghurt produced dramatic changes in its color [19]. In fact, as previously mentioned, elderberry juice is commonly used as a colorant source in foods such as dark fruit yoghurts, which it gives a red-purple characteristic color. Under the typical acidic conditions of yoghurts, the expected color of elderberry is reddish-purple, and this explained the color changes induced by the reformulation, which agreed with the higher a* (redness) values and the lowest luminosity (L*) and yellowness (b*) values observed by these authors [19]. Moreover, it is also important to highlight that the color was stable during storage (29 days), and no significant differences were observed in any of the color parameters. The lack of color differences demonstrated the high stability of the elderberry anthocyanins, or the lack of color differences could also be due to the condensation with other phenolic compounds, which resulted in other polymeric pigments [19].

In contrast, texture parameters showed no significant differences, but the elderberry yoghurt had the lowest values of firmness, consistency, cohesiveness, and index of viscosity. Thus, although the use of puree limited the effect observed with the direct addition of juice, the lower values of reformulated yoghurts could be related to the disruption of protein matrix integrity, which determines the gel structure and textural properties of yoghurt.

Regarding sensory analysis results, the authors did not find significant differences between natural and elderberry yoghurt treatments and in any of the storage days (days 1, 15, and 29). This fact indicated that the use of elderberry fruit puree is suitable for manufacturing a yoghurt with acceptable color, taste, odor, consistency, and general appearance [19]. Thus, elderberry puree was successfully used as a functional ingredient (increase bioactive compounds and dietary fiber), while improving the color of probiotic yoghurts without affecting either the texture or sensory quality.

The final study in which elderberry was used to reformulate yoghurt was conducted by Nemetz et al. [39]. Unlike the other studies, these authors used pomace powder collected after obtaining elderberry juice. Additionally, the “control” samples were formulated with commercial dyes, which included both, black carrot and purple sweet potato pigments, but no “natural” yoghurt (without colorants) was manufactured. After analyzing the elderberry yoghurts (containing 2% of elderberry pomace powder), these authors found that the total anthocyanin content stayed stable during yoghurt storage (14 days), which reflected the high stability of the anthocyanins of elderberry pomace powder. Moreover, this stability was higher in the yoghurts formulated with elderberry powder than those made with reference substances (black carrot and purple sweet potato pigments). A possible explanation for this behavior could be that in elderberry powder, the anthocyanins are bound to the intact cell structures and are progressively released during storage (14 days) [39]. This fact also explains the results of total phenolic compounds content, which increased slightly in elderberry yoghurts during storage and was due to the progressive release of these compounds from powder to yoghurt. Therefore, non-extractable polyphenols, which interact with cell wall material, were responsible for this gradual increase during the yoghurt storage [39].

In contrast with the previously discussed research, in this study, the use of elderberry pomace powder did not considerably change the color of reformulated yoghurts. In this case, the controls were formulated with commercial dyes, and only an increase in the Chroma values was observed. Therefore, elderberry pomace powder provided the same benefits as those with the addition of commercial dye (black carrot). In fact, the color stability of elderberry yoghurt was higher than those formulated with reference substances, which demonstrated the benefits of using elderberry pomace powder. Moreover, in addition to the color stability, the use of elderberry pomace also showed a potential health-promoting effect [39]. However, although the authors reported that elderberry pomace powder has promising techno-functional properties, its application in low viscosity foods could be difficult because of its high sedimentation velocity. They also concluded that the content of this powder could influence both, techno-functional and sensory properties of the reformulated food, thus a specific formulation should be tested and proposed for each food [39].

Kefir, another dairy product, was also reformulated with elderberry juice (10%) [23]. The authors tested kefir batches manufactured with different sweeteners. As occurred in the yoghurts, the addition of elderberry to kefir formulation produced a product with a reddish color. No differences were observed among batches since all of them used the same elderberry amount. This color was expected since anthocyanins present a red color in acidic conditions. In addition, the use of commercial elderberry juice resulted in kefir with a moderate amount of total phenolic compounds and a low amount of anthocyanins. The authors attributed this fact to the processing steps (thermal treatment, filtration, etc.) for obtaining elderberry juice, which could degrade the anthocyanin content [23]. Therefore, the authors reformulated another kefir batch using “fresh” elderberry juice. In this case, kefir manufactured with fresh juice had a more saturated color and 16-fold increase in anthocyanins content and 2-fold higher polyphenols content than in kefir with commercial juice [23]. This also produced a significant increase in the kefir’s antioxidant capacity.

In this study, kefir reformulated with elderberry juice and sucrose was well accepted by consumers (5.7% sucrose-sweetened kefir was the best accepted, followed by the 4.3% sucrose-sweetened kefir), while the use of non-nutritive sweeteners was not well accepted in the sensory analysis tests [23]. These results demonstrated that the acid profile of kefir in combination with the sweet taste of sucrose and the complex elderberry flavor contributed to obtaining a product with an excellent flavor that increased its acceptability.

Only two research studies were found on the use of elderberry in the meat industry. One of them evaluated the effect of elderberry vinegar (3%) (and other kinds of vinegar) on polycyclic aromatic hydrocarbons (PAH) formation in charcoal-grilled pork loin [47]. According to their results, spraying meat samples with elderberry vinegar exhibited the highest PAH inhibition (82%) in comparison with the other kinds of vinegar and control samples (without and with blank vinegar). In this study, the elderberry vinegar had the highest total phenolic compounds and also the highest antioxidant activity. This fact could explain the inhibitory effect since PAH formation involves free radicals generation; thus, the elimination of free radicals precursors with elderberry vinegar effectively reduced the PAH amounts in cooked meat. Therefore, as these authors concluded, spraying meat with elderberry vinegar prior to grill is an easy-to-apply strategy for limiting exposure to PAH [47].

In the other study, the researchers studied the effect of multiple plants, including elderberry powder (1%) as natural antioxidant in patties [52]. In this case, although the patties reformulated with elderberry powder showed higher peroxide values in comparison with control after 10 days of storage, the use of elderberry effectively decreased the formation of secondary oxidation products (thiobarbituric acid reactive substances; TBARs). In fact, elderberry patties showed a TBARs value that was less than half of that of control samples. Hydroperoxides are unstable, and in advanced stages of oxidation, the process of decomposition of hydroperoxides is greater than that of formation, so a decrease in the content of hydroperoxides was observed [2]. Thus, a low peroxide value may represent both, early and advanced oxidation [2]. This could explain the higher peroxide values in elderberry patties (early oxidation phase) in comparison with control samples (advanced oxidation phase). Once again, the high content of natural antioxidants in elderberry powder is the main reason for this behavior, since it delays lipid oxidation by multiple mechanisms, including by donating a hydrogen atom, binding metal ions or/and scavenging free radicals [52].

Unfortunately, none of the studies made in meat included the effect of reformulation with elderberry on the color, nutritional value, or sensory quality. Therefore, and taking into account the excellent properties of elderberry as an antioxidant and colorant, more studies are needed to investigate their impact on these parameters.

Bakery is another potential industry to use elderberry products. In a study, the authors proposed the use of elderberry powder (from 1% to 5%) as a natural colorant for the manufacture of gluten-free wafer sheets [50]. The authors investigated the effect in both the batter and in the final wafer sheets. The first beneficial effect observed was that the addition of elderberry powder increased more than 15-fold the bioactive compounds (flavonoids) content. Additionally, the use of elderberry also produced an enrichment in minerals (iron, potassium, calcium, magnesium, and sodium) [50]. In the batter, the use of elderberry powder produced lower batter delamination than control, which can be explained by the presence of pectin which, in combination with water, produces gelation. In contrast with other studies, the elderberry reformulated wafers presented lower a*, b*, and L* values with the increasing amount of powder used. The authors attributed this behavior to the color of the fruit powder used as an additive [50], and concluded that elderberry powder contains more blue pigments that impact the baked wafer color.

Regarding texture, the use of elderberry powder resulted in an increase in wafer breaking properties, which could be related to the higher fiber content in these wafers. Additionally, this parameter increased with growth in the content of elderberry, which agrees with the explanation of the effect of fiber content on the increase in breaking properties. Finally, after a sensory analysis, the wafers elaborated with greater elderberries powder (3–5%) presented the highest acceptable appearance scores. In addition, the most acceptable gluten-free wafers could be obtained by adding 5% of elderberry powders [50].

Similarly, other authors also proposed the use of different amounts of elderberry juice (2, 4, and 8%) as a coloring ingredient in croissants [6] and compared their results with two types of control (without dye and with commercial carrot dye). Although small (significant) modifications were observed in some parameters of the chemical composition of the croissants (reduced fat content, increased proteins, modified free sugars content, and produced changes in some fatty acids amounts), these were minimal, so it can be concluded that the reformulated croissants with elderberry maintained the nutritional quality of the original product [6].

Regarding color, a significant and progressive reduction in both L* and b* was observed with the elderberry juice inclusion, while the a* values increase. However, in the case of a*, this parameter did not change with the addition of 4% and 8% juice, which indicates that the addition of 4% of elderberry juice produced the same appearance change as that obtained using 8% juice [6]. Additionally, the antioxidant activity of the reformulated croissants was higher than control samples, but this antioxidant capacity was significantly reduced during the baking process, maybe due to the degradation of the bioactive compounds. The authors concluded that elderberry juice acted as a natural dye and a functionalizing agent, which added quality to the croissants. Additionally, they highlighted that elderberry provided the same benefits as those achieved with a commercial colorant (black carrot), demonstrating the potential of elderberry to be used in the food industry [6].

Finally, another study proposed the use of elderberry juice concentrate in three fiber-enriched kinds of pasta (methoxyl pectin, high methoxyl pectin, or Hi-maize starch) [51]. The first important aspect was that the addition of elderberry did not influence the pliability and processability of pasta. According to the authors’ observations, all dried raw pasta presented a good appearance and pleasant aroma. Regarding color, the elderberry addition produced a dark-purple color in raw dried pasta. This color was retained after cooking, which demonstrated the retention of elderberry anthocyanins [51]. In fact, the total antioxidant capacity and also the total polyphenol content increased with the addition of elderberry juice, both in raw dried and cooked pasta [51]. The authors reported that about 70% of antioxidant activity was retained after cooking the high methoxyl pectin pasta [51].

The inclusion of elderberry juice concentrate produced a significant decrease in firmness and an increase in cooking losses. The firmness of cooked pasta is related to the hydration of starch granules and their gelatinization in the protein matrix during the cooking process. Thus, the results suggest that elderberry could weaken the network formed by the gluten proteins, starch, and non-starch polysaccharides, and resulted in a reduction in the degree of starch gelatinization [51]. As a general conclusion, these authors noted that the use of elderberries improved the nutritional value of pasta and also presented acceptable quality characteristics.

## 4. Conclusions

Elderberries (and their products) are potential coloring agents and antioxidants that can be used in the food industry. However, despite the excellent coloring properties due to their high anthocyanin content, as well as their high antioxidant capacity (polyphenols, vitamins, etc.), nowadays, their use is rare, and there have been a very limited number of applications; thus, this product is underused.

Studies published on different foods report an obvious color change and also an increase in the antioxidant capacity of the food with the incorporation of elderberry products in the formulation. In addition, bioactive compounds are incorporated, which not only help to improve the shelf-life of food but also have important benefits for the health of consumers.

Even so, within the few studies that used elderberry for food reformulation, there are multiple ways to add it (juice, powder, pomace powder, concentrate, vinegar, etc.), making the results less comparable. This, together with the diversity of foods (dairy, meat, bakery, etc.) means that the addition of elderberries can have different implications. Therefore, it should be noted that more studies should be carried out on the application of *Sambucus nigra* berries as a coloring and antioxidant ingredient, as well as studies of their implications for the nutritional and sensory quality and the shelf-life of foods. In addition, and in those foods that are subjected to any treatment that can significantly affect the stability of the bioactive compounds provided by elderberry, stabilization techniques (e.g., encapsulation) should be used to increase their stability.

As a general conclusion, the incorporation of elderberry products has potential applications in the food industry since the use of elderberry is a promising strategy for producing functional foods and increasing their shelf-life. Moreover, the use of elderberries can help reduce the use of synthetic additives in food formulation. However, studies on the amount and the strategy used for elderberries’ incorporation should be carried out to ensure a positive effect on the nutritional and technological properties of foods, while improving or at least not affecting the sensory quality of the foods.

## Data Availability

All data are available in the manuscript.
